# Effects of a Health Education and Research Participation Enhancement Program on Participation and Autonomy in Diverse Older Adults

**DOI:** 10.1177/2333721420924952

**Published:** 2020-06-12

**Authors:** Allison A. Bay, Lindsay Prizer, Ahauve Orusa, Ariel R. Hart, Molly M. Perkins, Madeleine E. Hackney

**Affiliations:** 1 Division of General Medicine and Geriatrics, Department of Medicine, Emory University School of Medicine, Atlanta, GA, USA; 2 Emory University School of Nursing, Atlanta, GA, USA; 3 Birmingham/Atlanta VA Geriatric Research Education and Clinical Center, Decatur, GA, USA; 4 Atlanta VA Center for Visual and Neurocognitive Rehabilitation, Decatur, GA, USA; 5 Department of Rehabilitation Medicine, Emory University School of Medicine, Atlanta, GA, USA

**Keywords:** participation, autonomy, independence, older adults, family role, social role, quality of life, senior living, aging, race, diversity

## Abstract

Social engagement and autonomy are vital for life satisfaction among older adults. We measured multiple domains of social participation and autonomy in 120 adults over age 55 years that were part of an educational program at pretest, posttest, and follow-up. Quantitative and qualitative data were analyzed for differences between Black and White participants. White participants reported worse engagement in the family role domain and a lower Total Participation Score at posttest than pretest; however, scores returned to baseline levels by follow-up. Black participants reported better levels of participation in the social relationship domain at follow-up than at pretest. We found no evidence of qualitative differences between racial groups. Barriers to social participation and autonomy included challenges related to health, ageism, transportation, and mobility. Adequate housing, social support, socialization, and perception of individual utility contributed to feeling independent. Educational programs for older adults may provide an opportunity for increased social participation.

## Introduction

Participation, a construct encapsulating communication, mobility, interpersonal interactions, and self-care, allows fulfillment of valued life activities and social roles (Sau et al., 2015; Tomioka et al., 2016). The World Health Organization’s International Classification of Functioning, Disability, and Health (ICF) emphasizes the importance of social participation among all disabled individuals, including older adults who often contend with motor and sensory impairments. Autonomy refers to one’s ability to make freely self-directed choices in one’s life (Matsui & Capezuti, 2008) and is therefore often a precursor to participation. Participation/autonomy are associated with physical functioning, life-space mobility, and mood (Fallahpour et al., 2011; Portegijs et al., 2014). In the International Classification of Functioning, Disability, and Health (ICF) model, an individual’s functioning in activities is an interaction between health condition, environmental, and personal contextual factors (Kostanjsek, 2011). Understanding this interaction is relevant for clinical practice because one must consider demographics, the physical/social environment, and behavioral and psychological factors of the patient (Tomandl et al., 2018).

Studies indicate that in disabled and/or aging individuals, participation/autonomy are positively correlated with other well-being-related outcomes (Barkay & Tabak, 2002; Matsui & Capezuti, 2008; Mowad, 2004). In a study of individuals with late effects of polio, participation/autonomy were significantly associated with life satisfaction (Lund & Lexell, 2009). In older adults, increased participation is positively associated with higher health-related quality of life (Lantz et al., 2012). Social participation is positively associated with better functional skills, well-being, health-related quality of life, and survival in older adults (Dahan-Oliel et al., 2008).

Similarly, perceptions of aging and disease-related issues have a critical impact on participation/autonomy in older adults (Zafar et al., 2017). Perceived autonomy, specifically in participation outdoors, is more restricted among individuals with frailty and prefrailty (Portegijs et al., 2014). Ageism, the marginalization of older adults as a socially constructed state maintained by dominant ageist values in society (Braithwaite, Lynd-Stevenson et al. 1993), remains an inherent threat to independence and participation among older adults in a society that values youthfulness (Plath, 2007).

In addition, race and ethnicity are important factors to consider for participation levels of the individual, as sociodemographic factors influence barriers and facilitators of participation. Several studies suggest variations in perceptions of participation/autonomy among different racial and ethnic groups. Matsui & Capezuti (2008) found that White race compared with non-White was positively correlated with perceived autonomy among seniors (Matsui & Capezuti, 2008). In another study, a sample of 800 seniors found that Korean Americans and Mexican Americans tend to believe that family members should have a greater role in medical decision-making, while Europeans and African Americans felt that the individual should have more autonomy in medical decision-making (Blackhall et al., 1995).

Several factors may be related to racial differences in participation and autonomy in a variety of settings (Jackson & Erving, 2020). In research and medical settings, there is evidence that Black and African American individuals may be less likely to participate in research than White individuals based on a lack of trust in the medical and research communities (Shavers et al., 2002). In social settings, the experience of racism may result in social roles having differential impacts on individuals belonging to minority racial groups such that these individuals do not reap as many benefits of social participation, such as improved psycho-social health, as do members of racial majorities (Jackson, 1997). In an occupational setting, participation in a work role is linked to increased social capital, which can perpetuate wage inequalities between White and non-White populations (Smith, 2000). In addition, racial and ethnic minorities must often attempt to overcome personal barriers such as English language proficiency (Wolff & Ellis, 2009) and structural barriers such as unequal access to housing (Williams & Collins, 2001) to participate fully and enjoy autonomy in American society.

Sex association with participation in life activities depends on the domain. Anaby et al. (2009) examined personal and environmental factors in relation to participation among 200 older adults and found that sex played a significant role on participation in daily activities but not on participation accomplishments or social roles; however, this same study suggested that women tend to be more independent in daily activities and less independent in social roles (Anaby et al., 2009). Another study found that men tend to perceive less autonomy in their family role, possibly due to societal expectations (Fallahpour et al., 2011). Sex differences in participation have been primarily examined in specific activities including ultramarathon running (Senefeld et al., 2016), participation in depression trials (Gallo et al., 2005), and rowing (Keenan et al., 2018) rather than examining sex influence on participation overall.

The interaction between race and sex in relationship to participation/autonomy has not been thoroughly examined; however, such an interaction may well exist. In a 2018 survey of members of the Society for Epidemiological Research (SER), women who were racial or ethnic minorities were less likely to participate in events initiated by SER (DeVilbiss et al., 2020). Another study examining social isolation differences between Black and White adults found that Black women were least likely to be married, Black individuals of both sexes were less likely to have many close friends or relatives, White men were the least likely to regularly attend religious services, and men of both races were less likely to be involved in clubs or group activities than women (Alcaraz et al., 2019). This same study found that race was a better predictor of social isolation than sex, with White men and women less likely to be socially isolated than Black men and women (Alcaraz et al., 2019).

**Figure 1.**
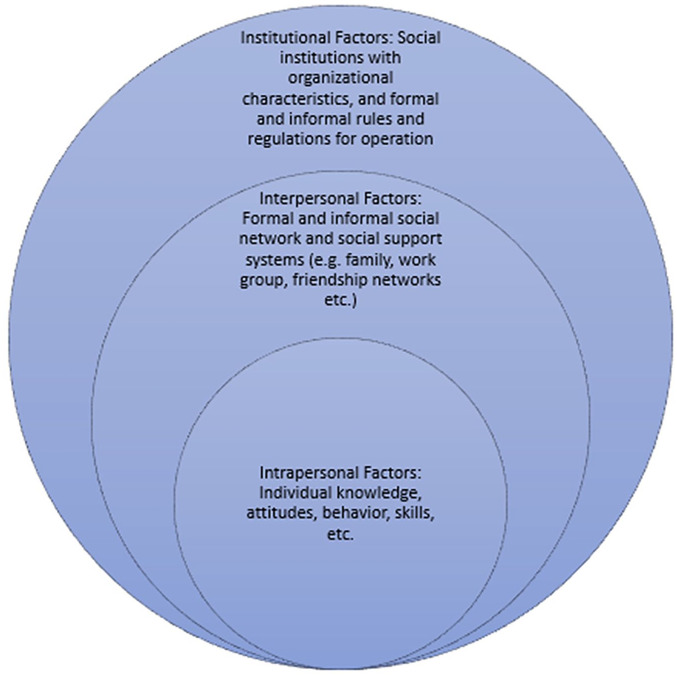
Conceptual model for factors that may be barriers or facilitators to participation and autonomy among older adults.

The conceptual model for this study is as follows: building upon the socioecological model (Kilanowski, 2017): intrapersonal, interpersonal, and institutional barriers exist for racial minorities in engaging socially in various contexts (Figure 1). Intrapersonal barriers include a lack of individual knowledge skills and distrust of authoritative professionals (medical, research, law enforcement, etc.), which may impede the individual’s ability to participate fully in all domains. Interpersonal barriers include overt racism, smaller formal and informal social networks, and social support systems resulting in reduced social capital. Institutional barriers to participation include disparities in wealth, income, and housing, health care and employment opportunities.

Similar barriers to participation and/or autonomy may exist for older adults with shrinking life-space and social networks. In addition to the barriers already mentioned, older adults may face aging and disease-related barriers. As such, an in-person 8-week-group educational program that brings together diverse individuals from a large metro area to learn together about aging-related topics that are relevant to all participants, regardless of race, sex, and ethnicity, may lead to enhanced participation in social spheres for all participants. Because the opportunities may be particularly unique for the Black individuals, it is expected they may benefit more from the opportunity.

While the relationship between participation/autonomy, health, and well-being is well-documented, no study has yet examined specific factors within participation/autonomy that may promote participation in community-dwelling older adults. Furthermore, it is not known what impact participating in a highly interactive health education program may have on participation/autonomy among this same group. Previous studies (Hart et al., 2017; Perkins et al., 2019) show that training designed to increase understanding of the research process is effective in increasing research participation. This study examined the impact of participation in the DREAMS program (Developing a Research Participation Enhancement and Advocacy Training Program for Diverse Seniors) on social participation by community-dwelling older adults.

We created the DREAMS program to prepare diverse older adults to engage in community-based participatory research and increase understanding of the research process and importance of participation while providing opportunities to participate in an interactive, two-part health education (Part 1) and research advocacy training program (Part 2). The DREAMS program is an 8-week education program focused on health and wellness with information about the research process embedded. Part 1 encouraged positive interactions with researchers in a lecture/discussion format. Study staff provided opportunities for participation in ongoing studies, community health education events, and recruitment fairs. Participants in Part 1 showed significantly less depression and satisfaction with the experience (Dillard et al., 2018). Those interested in receiving in-depth training about the research process could enroll in Part 2, an additional 8-week course on research processes and peer advocacy in research settings.

The DREAMS program was social in nature. The older adult participants were given opportunities to increase their social networks and exercise some of the better health practices that they learned about. Given the multiple opportunities afforded for increased participation and autonomy by this program, we hypothesized that participating in this health education program had the potential to improve social participation in their daily lives. In addition, we examined DREAMS’s effects on participation/autonomy in White versus Black older adults, because although the differences in opportunities and barriers for social participation are well-documented, nobody has yet compared participation levels in White and Black older adults within the context of an interactive health seminar designed to generate discussion and person-to-person engagement. We had three aims: (a) to examine independence and autonomy in a high-functioning, diverse sample of older adults, (b) to determine whether participation/autonomy significantly varies between older Black and White individuals, and (c) to examine differences in Impact on Participation and Autonomy Questionnaire (IPA) domain changes in response to DREAMS between Black and White participants. Qualitative methods were used to elucidate the factors that shape independence/autonomy.

## Methods

The Institutional Review Board at Emory University approved this work under protocol #80676. All participants provided written informed consent before participating. This manuscript details a subanalysis of a larger intervention-based study, described in depth in a separate paper (Dillard et al., 2018; Hart et al., 2017; Perkins et al., 2015, 2019).

### Participants

Adults age 55 years and over (*n* = 120) were recruited through presentations at local community events, flyers distributed in diverse senior living facilities, and word of mouth. Interested potential participants were contacted by phone to schedule initial assessments, and those who enrolled were assigned to an 8-week program of either in-person or at-home education. Of the 120 individuals who were recruited and completed the pretest, 103 individuals completed posttest assessments. As there were no statistically significant differences in outcomes between those who took the program in an in-person and at-home setting, results for all participants are presented. Those enrolled in the at-home program did not participate in the 1.5-hr classes but rather read the health education materials at home and then had 30-min phone calls every week with a research team member. These calls were intended to provide a proxy for social interaction and support, ensure fidelity to the program, and to assist participants with comprehension of the learning material. Both the in-person and at-home programs involved time for extensive person-to-person conversations.

### Baseline Health and Sociodemographic Measures

Trained raters administered measures according to standard procedures. A project health questionnaire was administered in the form of a survey to collect sociodemographic information. The Composite Physical Function (CPF) Scale measured participants’ abilities to complete activities of daily living (ADLs) (Jones et al., 2008). Single-item questions rated fall worry and quality of life. The frequency with which participants went out of their homes was also collected.

### Primary Outcome Measures

The primary outcome measure was the IPA, a reliable and valid instrument for assessing autonomy and participation (Fish, 2011). The IPA measures self-perceived participation in five domains: autonomy indoors, autonomy outdoors, social life, family role, and work/education, and includes a total score (lower is better). Participants were administered the IPA at three different times: within 1 week before (pretest), 1 week after (posttest), and 8 weeks after (follow-up) the intervention. Each subsection of the IPA includes the opportunity for participants to elaborate qualitatively on the topic (147 lines pretest, 140 lines posttest, 78 lines follow-up).

## Analyses

### Quantitative

We provide descriptive statistics including frequency, percentage, and mean values to summarize variables. Data were collected by repeated measures within the same subjects and outcome variables of IPA domain scores were ordinal. Because we needed to adjust for correlations between observations, and we were specifically interested in assessing if the change in average IPA differed between Black and White individuals (i.e., race, which is a fixed effect) at the sample level rather than individual level, a Generalized Estimating Equation (GEE) Poisson model was employed.

Race (Black and White), time point (pre, post, follow-up), and the interaction term between race and time point were included in the model. In the preliminary analysis, we confirmed that outcome variables did not show linearity with respect to time. Therefore, we calculated post/pre and follow-up/pre ratios (rather than linear slope) with 95% confidence interval (CI) to evaluate how each outcome changed over time. All analyses were conducted using R Studio (R Studio Teal, 2015) and “gee” package version 4.13 (Carey et al., 2015).

### Qualitative

As we did not find evidence for changes in themes among open-ended responses over time, qualitative responses to IPA were compressed across time points for each participant. Responses were reviewed for patterns and recurring themes. Emergent themes were considered in context of documented themes in pertinent literature related to health-related quality-of-life, social roles and support, and age-related risks to participation and autonomy. With consensus from the research team, final themes were condensed, refined, and developed (Vaismoradi et al., 2013).

### Triangulation of Quantitative and Qualitative Data

This is a mixed methods study that includes open-ended questions as part of the survey to inform and provide additional context for the quantitative survey findings. Consistent with this approach, data collection, analysis, and interpretation of findings occur simultaneously with equal priority and insights drawn from the combined methods presented in sections “Discussion” and “Conclusion.” We acknowledge that a survey study that includes open-ended questions as a simultaneous supplementary component to the quantitative survey does not provide the rich in-depth qualitative data characteristic of other mixed methods approaches. However, this approach does represent an accepted and commonly used mixed methods approach that can help validate and explain quantitative findings (Creswell & Plano Clark, 2011; Morse & Niehaus, 2009).

## Results

### Quantitative

This analysis included all participants with at least one observation (*n* = 120; Black *n* = 54, female = 41; White *n* = 66, female = 36; Table 1). Participants were approximately 70 years old. White participants (70.6 ± 9.4 years) were older than Black participants (65.6 ± 7.4 years). Participants on average had at least a bachelor’s degree. White participants had slightly more education than Black participants (16.36 and 15.21 years on average, respectively). Participants primarily lived independently (75.63% of sample), mostly drove their own vehicles (79.83% of sample), and were retired (81.51% of sample). Participants had similar numbers of comorbidities (average number= 3.08) and prescription medications (average number = 4.25), were equally likely to use an assistive device (no = 74.79%, yes = 15.97%, sometimes = 9.24%) and were relatively well able to perform ADLs as per the CPF (average score = 20.03/24). Most participants (54.62%) left the house daily. White participants were more worried about falls than Black participants (*p* = .040).

**Table 1.** Participant Characteristics.

Characteristics	Total (*N* = 120)	African American (*N* = 54)	White (*N* = 66)	*p*-value
	*M* (*SD*)/*N* (%)	*M* (*SD*)/*N* (%)	*M* (*SD*)/*N* (%)	
Sex^a^				.021^b^
Female	77 (64.17%)	41 (75.93%)	36 (54.55%)	
Male	43 (35.83%)	13 (24.07%)	30 (45.45%)	
Age (years)^c^	70.64 (9.36)	65.61 (7.44)	74.76 (8.77)	<.001^b^
Educations (years)^c^	15.85 (2.27)	15.21 (2.20)	16.36 (2.22)	.005^b^
Marital status^a^				.053
Single	15 (12.61%)	11 (20.75%)	4 (6.06%)	
Married	56 (47.06%)	24 (45.28%)	32 (48.48%)	
Other^d^	48 (40.34%)	18 (33.96%)	30 (45.45%)	
Housing^a^				.755
House/Apt/Condo	90 (75.63%)	42 (79.25%)	48 (72.73%)	
Senior housing	27 (22.69%)	10 (18.87%)	17 (25.76%)	
Other^e^	2 (1.68%)	1 (1.89%)	1 (1.52%)	
Transportation^a^				.360
Drive own vehicle	95 (79.83%)	40 (75.47%)	55 (83.33%)	
Other^f^	24 (20.17%)	13 (24.53%)	11 (16.67%)	
Occupational status^a^				.479
Employed	22 (18.49%)	8 (15.09%)	14 (21.21%)	
Not employed^g^	108 (81.51%)	45 (84.91%)	52 (78.79%)	
Years retired^b,c^	11.07 (9.33)	8.32 (7.31)	13.22 (10.21)	.014^b^
Number of comorbidities^c^	3.08 (2.19)	3.10 (2.42)	3.06 (2.01)	.932
Use assistive device for walking^a^				.170
No	89 (74.79%)	41 (77.36%)	48 (72.73%)	
Yes	19 (15.97%)	10 (18.87%)	9 (13.64%)	
Sometimes	11 (9.24%)	2 (3.77%)	9 (13.64%)	
Number of medications^c^	4.25 (3.54)	3.58 (3.01)	4.78 (3.85)	.064
Falls in previous year^c^	1.03 (2.81)	0.94 (2.39)	1.09 (3.13)	.769
Fall worry^c,h^	2.51 (1.37)	2.22 (1.44)	2.74 (1.28)	.040^b^
Self-rated quality of life^c,i^	5.46 (1.24)	5.30 (1.46)	5.58 (1.03)	.238
Composite physical function score (/24)^c^	20.03 (4.84)	20.02 (4.91)	20.05 (4.82)	.976
Frequency of leaving house^a^				.031^b^
<1 Per week	1 (0.84%)	1 (1.89%)	0 (0.00%)	
1–2 Times per week	8 (6.72%)	6 (11.32%)	2 (30.30%)	
3–4 Times per week	45 (37.81%)	24 (45.28%)	21 (31.82%)	
Everyday	65 (54.62%)	22 (41.51%)	43 (65.15%)	

*Note.* Characteristics of participants. Frequency of different characteristics may vary because of missing data. Missing data were excluded when calculating percentage, mean, and *SD*.

a Chi-square tests or Fisher’s exact tests were used for categorical variables. ^b^Excluding those who have not retired or missing data. ^c^Welch Two sample *t*-test was used for continuous variables. ^d^Includes Separated/Divorced, and Windowed. ^e^Includes assisted living, relative homes, and others. ^f^Includes family/friends drive, transportation service, and public transportation. ^g^Includes homemaker, retired, volunteer, and disability. ^h^Treated as continuous, out of 7, lower is better. ^i^Treated as continuous, out of 7, higher is better.

Postintervention, 52 White participants and 48 Black participants had completed the intervention. Performance on IPA by group and time point is presented in Table 2. At the posttest time point, four Black and 13 White participants who had participated in pretests did not attend posttest sessions and were therefore considered “Withdrawals.”

**Table 2.** Quartile Scores for IPA Domains and IPA Total at Pre, Post, and Follow-up Time Points.

Group	Pre						Post						Follow-up					
	*N*	*Mdn* Q2	25% Q1	75% Q3	*M*	*SD*	*N*	*Mdn* Q2	25% Q1	75% Q3	*M*	*SD*	*N*	*Mdn* Q2	25% Q1	75% Q3	*M*	*SD*
Black																		
IPA total	54	13.5	2	28.0	18.31	19.3	50	18.5	1.25	32.0	19.68	18.5	48	9	0	27.3	16.71	19.1
Autonomy indoors	54	0	0	1	1.35	2.78	50	0	0	2	1.58	2.73	46	0	0	1.75	1.39	2.75
Family role	54	1	0	6	3.41	4.29	50	2.5	0	6	3.56	3.91	46	2	0	5	3.2	4.28
Autonomy outdoors	54	2	0	5	3.06	3.37	50	3	0	5	2.96	3.14	46	1	0	5	2.89	3.53
Social relationships	54	3.5	0	6	3.87	4.12	50	2	0	5	3.74	4.16	46	1	0	6	2.98	3.77
Work and education	54	3	0	5	3.69	4.43	50	2.5	0	7	4.16	4.8	46	1.5	0	6	3.61	4.97
Problem score	54	2	0	5	2.94	3.67	49	2	0	7	3.29	3.76	46	0	0	6	2.87	3.77
White																		
IPA total	66	10.5	4	24.8	17.77	18.9	53	17	4	33.0	21.28	20.6	52	9	3.75	25.0	17.79	20.72
Autonomy indoors	66	0	0	0	1.21	2.96	53	0	0	2	1.49	2.74	52	0	0	1	0.98	2.3
Family role	66	1	0	5	3.02	4.36	53	1	0	6	3.64	4.75	52	1	0	5	3.15	4.43
Autonomy outdoors	66	2	1	5	3.33	3.62	53	3	0	5	3.3	3.77	52	1.5	0	4.25	3.17	4.02
Social relationships	66	3	1	6	3.74	3.52	53	3	0	7	4.38	4.67	52	3	0	7.25	4.19	4.79
Work and education	66	3	0.25	6	4	4.63	53	4	0	8	5.02	5.02	52	3	0	5	4.12	4.71
Problem score	66	1	0	4	2.53	3.38	53	2	0	6	3.13	3.23	52	1	0	4.25	2.5	3.24

*Note.* Quartile scores for IPA domains and IPA total at pre, post and follow-up time points by race. IPA = Impact on Participation and Autonomy Questionnaire.

Table 3 presents IPA differences by group across time. White participants reported worse levels of participation in the domains of Family Role (95% CI = [1.027, 1.529]) and Total Score (95% CI = [1.036, 1.383]) at posttest than at pretest; however, these scores returned to baseline levels by follow-up. Black participants reported better levels of participation in the Social Relationship domain at follow-up than at pretest (95% CI = [0.589, 0.947]).

**Table 3.** Differences in IPA Domains^a^ Across Time.

Group	Variable	Pre	Post	Follow-up	Post/Pre proportion		Follow-up/Pre proportion	
		*M* ^b^	*M* ^b^	*M* ^b^	Estimate	Robust [95% CI]	Estimate	Robust [95% CI]
Black	IPA total	18.315	19.274	15.911	1.052	[0.854, 1.297]	0.869	[0.720, 1.049]
	Autonomy indoors	1.352	1.541	1.383	1.140	[0.664, 1.957]	1.023	[0.607, 1.663]
	Family role	3.407	3.473	3.105	1.019	[0.785, 1.323]	0.911	[0.710, 1.1700]
	Autonomy outdoors	3.056	2.902	2.765	0.950	[0.749, 1.204]	0.905	[0.740, 1.106]
	Social relationships	3.870	3.706	2.890	0.958	[0.770, 1.190]	0.747	[0.589, 0.947]*
	Work and education	3.685	4.114	3.558	1.116	[0.762, 1.636]	0.966	[0.657, 1.419]
	Problem score	2.944	3.173	2.913	1.078	[0.828, 1.404]	0.989	[0.727, 1.342]
White	IPA total	17.751	21.243	17.588	1.197	[1.036, 1.383]*	0.991	[0.850, 1.155]
	Autonomy indoors	1.212	1.501	0.972	1.239	[0.867, 1.770]	0.802	[0.528, 1.219]
	Family role	3.015	3.778	3.247	1.253	[1.027, 1.529]*	1.077	[0.858, 1.353]
	Autonomy Outdoors	3.325	3.317	3.212	0.997	[0.838, 1.187]	0.966	[0.814, 1.146]
	Social relationships	3.742	4.197	3.985	1.121	[0.923, 1.362]	1.065	[0.871, 1.301]
	Work and education	3.993	5.018	4.078	1.257	[0.939, 1.682]	1.021	[0.764, 1.366]
	problem score	2.530	3.129	2.466	1.237	[0.968, 1.580]	0.975	[0.767, 1.239]
Total	IPA total	18.006	20.270	16.758	1.126	[0.993, 1.277]	0.931	[0.824, 1.051]
	Autonomy indoors	1.275	1.517	1.163	1.190	[0.861, 1.643]	0.913	[0.659, 1.264]
	Family role	3.191	3.615	3.167	1.133	[0.959, 1.338]	0.992	[0.837, 1.177]
	Autonomy outdoors	3.204	3.122	3.004	0.974	[0.844, 1.124]	0.937	[0.822, 1.069]
	Social relationships	3.800	3.963	3.471	1.043	[0.901, 1.208]	0.913	[0.777, 1.073]
	Work and education	3.855	4.583	3.833	1.189	[0.940, 1.504]	0.994	[0.786, 1.258]
	Problem score^c^	2.717	3.139	2.669	1.156	[0.965, 1.385]	0.982	[0.808, 1.194]

*Note.* 8 Weeks lapsed between pre and post. 8 Weeks lapsed between post and follow up time points. CI = confidence interval; IPA = Impact on Participation and Autonomy Questionnaire.

a Questions are summed within each domain. Higher scores indicate less participation/autonomy in the domain. ^b^General Estimating Equation was used to estimate means. ^c^Problem Score is graded on a 3-point rating scale ranging from 0 (*no problem*) to 2 (*severe problem*).

* 95% CI does not cross 1, indicating significance in bold.

### Qualitative

Open-ended responses were provided by the following participants: 57 of 132 participants during pretest, 47 of 113 participants who had a posttest during posttest time point, and 33 of 108 participants who had a follow-up during the follow-up time point.

#### Race

Of the 74 participants who responded to questions qualitatively at any time point, 34% (*n* = 25) were Black or Other. Minimal differences by race were observed on any qualitative themes. Black and White participants responded at comparable rates to all themes.

#### Facilitators and inhibitors of participation and autonomy

Given that no significant differences by race or across time points were found, the following qualitative findings for all participants are presented by theme rather than by race or time point. Eight themes, either challenging or facilitating participation/autonomy, emerged. Factors negatively influencing participation/autonomy included health, transportation, mobility, and negative perceptions of aging. Facilitators of autonomy include housing, support, socialization, and a perception of individual utility. These themes are discussed in greater depth in the following section. Instrumental/social support was a barrier and a facilitator depending if the participant was giving or receiving this support.

#### Negative- Poor health

A variety of health issues were noted as affecting participation/autonomy. Memory, back pain, knee pain, and arthritis were the most commonly cited health issue noted, and general health was also noted frequently. There were 23 unique responses related to health challenges, affecting all but three of the 12 questions in the IPA. Health was most often cited as having a negative impact on participants’ mobility (Q1), ability to work (Q8), and participate in education (Q9). The following statements are representative of this theme:

- I have hearing loss which affects my getting jobs, all my life, but especially now at age 67.
- Not being able to do much walking or standing would limit the jobs I could get.
- I am limiting my driving now, making changes to accommodate . . . I have a hearing problem and it makes me anxious to travel alone.

#### Negative- Transportation issues

Twelve participants noted increasing limitations with driving, which several supposed were due to age. Others mentioned issues with air travel (*n* = 2) or with traveling/transportation generally (*n* = 5). Transportation issues affected seven of the 12 questions, highlighting the pervasive effects of impairment in this domain:

- Driving has become more of an issue; lack of confidence since I don’t drive as much when I worked.
- Driving long distances is becoming a problem.
- My children want me to quit driving because I am 80 years old.
- I wanted to visit my son who lives in Salt Lake City but because of vertigo I was afraid to fly.
- Minor problems because someone else drives me for long trips. I can only drive so far.

#### N﻿egative- Mobility Issues

Mobility was noted as separate from transportation, pertaining directly to an individual’s ability to walk, which significantly impaired several domains of participation/autonomy. Fifteen respondents cited issues in mobility, affecting four of the 12 IPA questions:

- I need assistance getting around to help [keep] me from falling.
- I am unable to move about on my own. I need a gait belt and someone to assist me at all times.
- Mobility is not good right now and [I] don’t know if it will come back.

#### Negative- Multiple issues

Often multiple themes intersected to create challenges in participation/autonomy. For instance, the following participant notes health issues that negatively affect mobility, subsequently limiting individual autonomy:

- I have back pain and a foot that needs surgery. It is difficult to stand very long or to walk very far.

#### ﻿Negative- Ageist perceptions directed at self or others

Eleven participants expressed concerns of ageism or projected ageism as having a negative impact on participation/autonomy on four questions:

- Most people don’t want people my age for voluntary work.
- I’m not really looking for work, but if I did, working in my field is not likely as there is such thing as age discrimination and “over qualification.”
- As an older person, one is not so accepted as a still valuable person . . .

#### Positive- Housing

Housing was mentioned as having a significant positive contribution to participation/autonomy. Most participants discussed amenities provided by condominium staff or by different levels of senior living facilities. Twelve participants noted on seven questions the importance of housing as a support for their independence. Only one participant mentioned housing as a negative, noting that the stairs were a barrier to being able to go into the basement when desired:

- I live in a retirement community where house helpers and maintenance people are included in [a] monthly fee.
- I live in a place where they have a maintenance team to do repairs and have no garden.
- I live in an independent living facility. I have a housekeeper once a week and a maintenance man when I need him.
- We live in a condo where help is provided.

#### Positive- Socialization

For several respondents, housing positively affected their ability to socialize, enhancing that domain of participation:

- [My facility] provides extensive social possibilities at the push of an elevator button.
- Many social activities are available at the condo.

#### Positive and negative- Instrumental and social support

Perception of support depended on if the participant were receiving or providing the support. Outside support from friends, family, and hired help was cited as a significant factor enabling participation/autonomy. Twelve participants noted the importance of outside support on four of the 12 IPA questions. Conversely, caregiving was mentioned 12 times as being a hindrance to other domains of participation:

- I rely on some outside help, ex: housekeeper and gardener.
- Although I cannot perform these tasks on my own, my family, friends, and supporters all make sure that my requests are met.
- John the handyman has many skills!
- I have responsibilities which do not allow me to go as I please, I am a caregiver.
- I provide care for a spouse who needs me around and that can limit my ability to go and come as I might choose.

#### Positive- Individual utility

Thirteen respondents emphasized the importance of feeling useful and helpful in remaining autonomous. The two pertinent questions asked about the individual’s participation in volunteer and paid work specifically:

- I do volunteer work at my church, and it’s a motivator for me to make the time and do it. Makes me feel useful and gives me joy.
- I would be very sorry if I could not help others in need.

## Discussion

The quantitative differences that emerged between Black and White participants highlight the need to examine the influence of individual characteristics on participation to improve autonomy among unique groups of older adults.

White participants reported less participation overall (sum of individual domains scores) and participation related to family role immediately after the program. This finding is perplexing. The overall decreased participation among the White participants was an unintended and unanticipated consequence of the program. Possibly this finding is a result of increased understanding of health and its impact on family, leading to the participant’s perception of not meeting standards. Similarly, it may reflect increased contemplation by the participants regarding their own health and wellness as pertaining to independence, leading to reduced participation in life’s activities. Family role is influenced by context, gender, marital status, and types of family leisure activities (Altergott & McCreedy, 1993), emotional relationships between family members, the diversity of family structures and households, the interdependence of family members on one another and individual members’ functions, and caregiving for one another (Silverstein & Giarrusso, 2010). Given the complexity and multi-dimensionality of one’s role within a family structure, possibly the family role for White participants was influenced by the DREAMS program. As both family role participation and total participation returned to levels similar to baseline levels at the follow-up time point, these negative consequences of the program appear to be short in duration.

The Black participants had no changes in total participation at posttest but displayed encouraging findings at follow-up indicating increased participation levels in social relationships. A study examining differences in social networks between Black and White older adults found that Blacks had smaller social networks, but had more contact with network members, and more family members in their networks (Ajrouch et al., 2001). If Black participants in our sample had smaller, more tight-knit social circles than the White participants, and new contacts made through the DREAMS program were incorporated into Black participants’ social networks, then the knowledge gained and new friendships created by the DREAMS program may have impacted the Black group more. The expanded social circles may help explain the observed increased Black participation in social relationships.

Even though Black participants were, on average, younger and female, we observed no differences racially in IPA at baseline. We might have expected to see more evidence of age-related changes in the White groups such as residing in independent living, less mobility, relying on others for transportation, more comorbidities, greater number of prescription medications, and greater use of an assistive device for walking. However, as participants in both groups were similar in these domains, it does not appear that age or sex differences between groups were significant factors in participation or autonomy at baseline, possibly because both groups were active and relatively healthy.

The influence the factor, sex may have exerted over participation and autonomy in this study must be interpreted with caution, as there were 30 White men and 13 African American men in this study. This difference in representation may have influenced observations between men and women on IPA domains in unknown ways. However, given that men tend to report less autonomy on the IPA in the family role domain (Fallahpour et al., 2011), perhaps the DREAMS program helped empower men to participate in their family role to a greater extent than women, who may have already had high participation. Although infeasible for this analysis due to the relatively nonnormality of the distribution of sex by race in our sample, future studies should attempt to equalize the number of participants by sex and race to examine the interaction of these factors on IPA domains.

Unsurprisingly, health emerged as a prominent theme that negatively impacted participation/autonomy in numerous domains when compromised as health risk factors increase with aging (Hodes et al., 2016). However, both projected and internalized ageist statements were unexpected. Additional research into internalized ageism could render important findings regarding quality of life, engagement, and self-perception in older age. Health-related stigma is well-documented across chronic conditions (Van Brakel, 2006), although it is not examined as widely at the intersection of aging. Although numerous types of stigma exist, perceived or internalized stigma includes “the devaluation, shame, secrecy and withdrawal triggered by applying negative stereotypes to oneself” (Boyd Ritsher et al., 2003; Van Brakel, 2006). Although none of the participants cited overt examples of discrimination, several expressed feelings of this internalized stigma as a potential deterrent to increased participation. Experiences of discrimination should be examined further to better understand their impact on participation, social activity, and autonomy in older cohorts.

We were surprised by the positivity of participants’ views toward senior independent housing. A study by Castle (2011) found that nine of 10 seniors would prefer to age in their own homes compared with assisted or independent living facilities (Castle, 2011). The qualitative comments provided by participants in this study suggest that in the context of health and aging, senior independent housing was perceived as enhancing participation/autonomy, particularly with respect to additional support and socialization opportunities for older adults.

### Limitations to the Study

The groups were different in age and sex distribution which may have affected the study findings. Women tend to report more loneliness than men, and older adults and those who are less educated tend to be more socially isolated than their counterparts (Tanskanen & Anttila, 2016), which could differentially influence responses on the IPA. However, participants were recruited from sources representing this segment of the metro Atlanta population. Furthermore, there may have been an association between age and race which influenced attrition. Black attrition (7.4%) was lower than White attrition (20%). Loss to follow-up among older adults is associated with older age, lower education, living alone, renting housing, and greater functional impairments, and is often nonrandom (Mihelic & Crimmins, 1997). Therefore, higher attrition among Whites may reflect that this cohort was, on average, older. We do not believe that age accounts for all observed differences as all participants were recruited via the same channels, suggesting that the younger Black adults were experiencing life circumstances comparable with older White adults.

White participants had an average of one additional year of education compared with Black participants; however, all participants had an average of a bachelor’s degree. Given that everyone was highly educated, these findings do not generalize to lower socioeconomic status (SES) groups. It is likely that the high education levels muted rather than exacerbated findings, as higher education can cause people to feel lonelier among less-informed peers (Tanskanen & Anttila, 2016). Individuals in this sample may have already been experiencing negative education-related impacts on participation, causing effects observed to be smaller than if the study had been conducted among a less educated cohort.

Finally, many of the participants did not provide any qualitative comments. As such, the themes that emerged from the thematic analysis only represent a small subsample of participants. Individual interviews and focus-groups among Black and White cohorts would further elucidate qualitative differences between these groups not captured in the current analysis.

## Conclusion

High levels of participation/autonomy across many realms are vital to maintaining a high quality of life among older adults. As poor health, transportation barriers, mobility limitations, and ageism were all identified by participants as factors that impede participation, every effort should be made to reduce these challenges. Educational programs tailored to older adults may provide an opportunity for increased social participation in the form of friendships and expand social networks. This effect may be more pronounced for Black participants, who may tend to have smaller, closer-knit social circles.
